# Fluoride Release from Pediatric Dental Restorative Materials: A Laboratory Investigation

**DOI:** 10.3390/dj13050224

**Published:** 2025-05-21

**Authors:** Angelo Aliberti, Roberta Gasparro, Maria Triassi, Mirko Piscopo, Pietro Ausiello, João Paulo Mendes Tribst

**Affiliations:** 1 Department of Neurosciences, Reproductive and Odontostomatological Sciences, University of Naples Federico II, Via Sergio Pansini 5, 80131 Naples, Italy; ange.aliberti@studenti.unina.it (A.A.); roberta.gasparro@unina.it (R.G.); mirko.piscopo.98@gmail.com (M.P.); 2 Interdepartmental Research Centre in Health Management and Innovation in Healthcare (CIRMIS), Via Sergio Pansini 5, 80131 Naples, Italy; maria.triassi@unina.it; 3 Department of Public Health, University of Naples Federico II, Via Sergio Pansini 5, 80131 Naples, Italy; 4 Department of Reconstructive Oral Care, Academic Centre for Dentistry Amsterdam (ACTA), Universiteit van Amsterdam and Vrije Universiteit Amsterdam, 1081 Amsterdam, The Netherlands; joao.tribst@gmail.com

**Keywords:** fluoride, glass ionomer cements, pediatric dentistry, dental materials, preventive dentistry, ions

## Abstract

**Objectives:** Dental caries remains a prevalent issue in pediatric dentistry, necessitating restorative materials that not only repair decay but also provide protective benefits. Fluoride-releasing restorative materials have a key function in preventing recurrent caries by inhibiting bacterial activity and promoting remineralization. The objective of this study was to examine fluoride release from three pediatric dental restorative materials—*Riva Light Cure HV*, *Fuji IX GP Fast*, and the *Cention Forte Filling Material*—under different pH and temperature conditions. **Methods**: Specimens (10 mm diameter and 2 mm thickness; n = 3 for each material) were prepared according to the manufacturers’ instructions; immersed in buffer solutions at pH 4.8, 6.8, and 8.8; and stored at 37 °C and 44 °C. Fluoride release was quantified using ion chromatography at three time points (1 day, 7 days, and 28 days). **Results**: The data revealed that fluoride release was significantly influenced by pH, temperature, and time (*p* < 0.05). *Riva Light Cure HV* exhibited the highest release, particularly in acidic conditions (pH 4.8), reaching 40.14 mg/L at 44 °C after 28 days. The *Cention Forte Filling Material* and *Fuji IX GP Fast* also showed increased release over time, but with lower cumulative concentrations. Higher temperatures generally enhanced fluoride diffusion across all materials. **Conclusions**: These findings emphasize the pivotal impact of environmental factors in fluoride release dynamics. *Riva Light Cure HV* demonstrated superior fluoride release, particularly in acidic environments, suggesting its potential for high-caries-risk pediatric patients. These insights can inform the selection of restorative materials in pediatric dentistry, optimizing caries prevention strategies.

## 1. Introduction

Childhood represents a critical phase in dentition development, during which deciduous teeth are particularly vulnerable to carious processes [1,2]. Dental caries remains one of the most widespread oral diseases globally, affecting 60–90% of both children and adults [3]. Among children, early childhood caries (ECC) is especially prevalent, with severe cases impacting children under three years old, often leading to significant complications in oral health and general well-being [4]. The management of dental caries in pediatric patients presents a unique challenge, requiring both preventive strategies and effective restorative solutions [5,6]. The assessment of dental caries’ incidence and severity is commonly carried out using epidemiological indices such as the DMFT (Decayed, Missing, and Filled Teeth) index for permanent dentition and the dmf-t index for deciduous teeth [7,8]. These indices provide essential tools for monitoring epidemiological trends and evaluating the effectiveness of preventive measures over time. The primary approach to caries management in pediatric dentistry emphasizes preventive strategies [9] aimed at reducing the incidence of caries, complemented by restorative treatments that eliminate carious lesions while preserving dental structure and function [10]. However, the durability of restorative materials and their ability to prevent secondary caries remain pivotal concerns [11].

Among the materials used in pediatric restorative dentistry, glass ionomer cements (GICs) are a cornerstone due to their biocompatibility, fluoride-releasing properties, and chemical bond to tooth structure [12,13,14]. These materials facilitate calcium complex precipitation at the tooth–restoration interface, also contributing to enamel remineralization. However, traditional GICs have some limitations, including prolonged setting times, susceptibility to moisture contamination, suboptimal mechanical strength, and aesthetic constraints [15,16]. To enhance the longevity and effectiveness of GIC-based restorations in children, resin-modified glass ionomers (RMGIs), a class of photocuring filling materials, were developed by incorporating resin components such as hydroxyethyl methacrylate (HEMA) to improve mechanical strength [17,18].

Recent developments in material science have led to the emergence of bioactive restorative materials that not only release fluoride but also demonstrate bactericidal and bacteriostatic properties. These materials facilitate the exchange of calcium and phosphate ions with the dental structure and support the remineralization of demineralized dentin [19,20,21]. Moreover, they enhance the natural adhesion between the restoration and the tooth, promote apatite formation on the surface, and exhibit self-sealing capabilities, thereby minimizing the risk of recurrent damage and secondary caries. Among the tested materials, the *Cention Forte Filling Material* exhibits bioactive properties due to its ion-releasing capacity, promoting remineralization and supporting self-sealing effects at the restoration margins [22].

Fluoride-releasing restorative materials, such as GIC and fluoride-containing composites, play a crucial role in preventing recurrent caries by inhibiting bacterial activity [23] and promoting remineralization [24,25]. However, the limited fluoride release from composite resins, due to the restriction of fluoride diffusion within the resin matrix, has led to investigations aimed at enhancing fluoride release. Modifications like the incorporation of fluoride-containing glass particles or hydrophilic monomers, such as 2-hydroxyethyl methacrylate, have been explored to improve fluoride release and its bioactive effects over time [26,27,28]. In pediatric patients, where compliance with oral hygiene practices may be inconsistent, the presence of fluoride-releasing restorations can serve as an adjunct to routine fluoride exposure through toothpaste, mouth rinses, and dietary sources [29].

Although the fluoride release dynamics of newer materials such as GICs, rmGICs, and alkasites like *Riva Light Cure HV* (SDI), *Fuji IX GP Fast* (GC), and the *Cention Forte Filling Material* (Ivoclar) have been studied, much remains to be understood about their behavior in the complex oral environment. Factors such as pH fluctuations and temperature variations can significantly influence fluoride release and rechargeability, making it essential to further investigate these interactions to optimize material performance [19].

This in vitro study aims to explore the short- and long-term fluoride release from various pediatric restorative materials (*Riva Light Cure HV*, *Fuji IX GP Fast*, and the *Cention Forte Filling Material*) under varying pH and temperature conditions. The research also aims to assess how specific environmental factors, such as pH and temperature, affect the fluoride release characteristics of these materials and influence their bioactive properties. By analyzing their remineralization potential and bioactive properties, this study seeks to contribute to the advancement of caries management and prevention strategies in pediatric dentistry, ultimately enhancing the outcomes for young patients. The null hypothesis states that all restorative filling materials release fluoride at the same rate, and that environmental pH and temperature have no impact on this release.

## 2. Materials and Methods

### 2.1. Sample Preparation

Three commercially available restorative materials commonly employed in pediatric dentistry were evaluated. The composition and key properties of each material are presented in Table 1. Specimen preparation was carried out in accordance with the manufacturers’ instructions. Specifically, the *Cention Forte Filling Material* (Ivoclar) and *Fuji IX GP Fast* (GC Europe) were prepared via self-curing techniques, while *Riva Light Cure HV* (SDI) was polymerized using light-curing. Stainless steel molds were used to shape the *Cention Forte Filling Material* and *Riva Light Cure HV* specimens, whereas a Teflon mold was used for Fuji IX GP Fast. All molds had a diameter of 10 mm and a thickness of 2 mm, which is consistent with the methodology described by di Lauro et al. (2023) [22] and Jaganath et al. (2024) [30]. Mixing was performed using a 3MTM ESPETM CapMixTM unit (3M ESPE, Seefeld, Germany) for 10 s, after which the materials were immediately transferred into the molds. A celluloid strip and a smooth condenser were used to gently compact the materials, aiming to eliminate air bubbles and achieve a uniform surface. No surface coating was applied. After a setting time of 5 min, the specimens were demolded and polished using 800-grit abrasive paper under water cooling with a rotating polishing system (Ecomet 30, Buehler Ltd., Lake Bluff, IL, USA).

### 2.2. Material Testing Conditions

The analyses were performed according to the protocol outlined by di Lauro et al. (2023) [22]. Briefly, samples (n = 3 per material) were immersed in 50 mL of buffer solutions at three distinct pH levels (4.8, 6.8, and 8.8) and incubated in temperature-controlled laboratory ovens (Precision Thelco, Thermo Fisher Scientific, Waltham, MA, USA) maintained at 37 °C and 44 °C. The higher temperature (44 °C) was chosen to replicate the transient elevation of intraoral temperature following the consumption of hot foods or beverages, as previously described in the literature [22,31,32].

For the acidic environment (pH 4.8), a 1M CH_3_COOH/CH_3_COONa_3_ × H_2_O buffer was employed; a phosphate–citrate buffer was used for the neutral condition (pH 6.8); and a 1M Tris-HCl buffer was prepared for the basic condition (pH 8.8). Specimens remained in their respective solutions for 1, 7, and 28 days before being transferred into 50 mL Falcon tubes for subsequent analysis.

#### 2.2.1. pH Measurement and Evaluation

For pH assessment and evaluation, 5 mL of the soaking solution was extracted from each sample and transferred to 15 mL Falcon tubes. A digital pH meter (Mettler Toledo Seven Excellence pH/Cond Meter S470-Std-K; Mettler-Toledo S.p.A., Milan, Italy)previously calibrated with standard solutions (pH 4.0, pH 7.0, and pH 9.0), was used for the measurement.

#### 2.2.2. Fluoride Release Evaluation

The cumulative release of fluoride was assessed using ion chromatography. A 1 mL aliquot of the soaking solution from each specimen (n = 3 per material) was collected and placed into 1.5 mL vials for analysis. Ion concentrations were measured with a DIONEX Integrion HPIC™ ICS1100 ion chromatography system (Thermo Fisher Scientific, Bremen, Germany) equipped with an IonPac AS27 RFIC™ analytical column (4 mm × 250 mm; Thermo Fisher Scientific). The analysis was carried out using a suppressed conductivity detector, which is standard for fluoride quantification due to its sensitivity and selectivity. The column temperature was maintained at 30 °C, and a 25 µL sample was injected at a flow rate of 1.0 mL/min. Ion concentrations were quantified based on the retention times of the chromatographic peaks, using calibration curves generated from standard fluoride solutions. Calibration curves were prepared with six concentration points: 1.0, 2.5, 5.0, 10.0, 25.0, and 50.0 mg/L.

### 2.3. Statistical Analysis

For the statistical analysis, the STATA 14.0 program (College Station, TX, USA) was employed. Descriptive statistics were performed to determine the mean concentrations, standard error, as well as minimum and maximum values for each material. The statistical significance of each independent variable was examined using the Wald test, with a significance threshold set at *p* < 0.05. The Shapiro–Wilk test was utilized to evaluate the normality of the variable distribution. Mixed-effects models (MEMs) were implemented to analyze intergroup differences based on pH, temperature, and exposure time. The statistical significance of each independent variable was examined using the Wald test, with a significance threshold set at *p* < 0.05.

## 3. Results

### 3.1. pH Measurements

**Figure 1:**
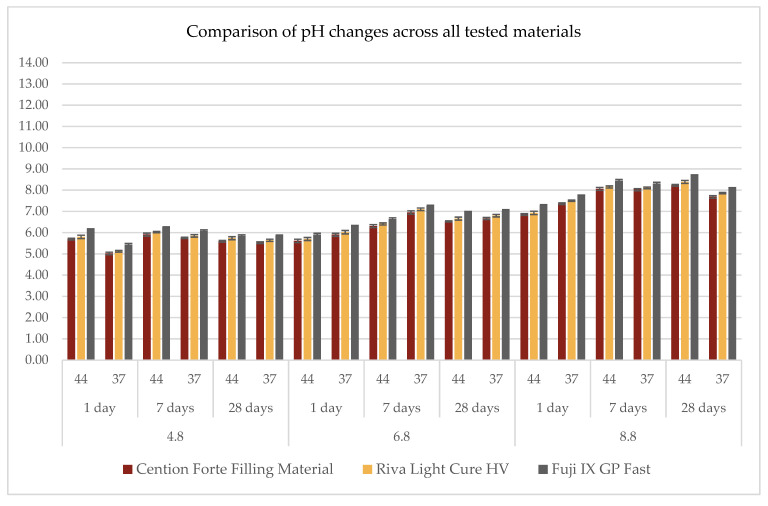
Comparison of pH variation for all three materials tested in three buffered solutions (pH 4.8, 6.8, and 8.8), at two temperatures (37 °C and 44 °C) and at three observation times (1 day, 7 days, and 28 days).

The analysis of pH variations across different materials under controlled experimental conditions—including acidic, neutral, and basic environments, at two temperatures (37 °C and 44 °C), and over three observation periods (1 day, 7 days, and 28 days)—is presented in Figure 1 and detailed in Table 2.

All tested materials demonstrated similar trends, showing only minor variations in pH values. In acidic conditions (pH = 4.8), a general increase of approximately one pH unit from the initial values was observed (Figure 1). Specifically, the *Cention Forte Filling Material* exhibited pH levels ranging from 5.02 ± 0.06 at 37 °C after 1 day to 5.91 ± 0.07 at 44 °C after 7 days, with an overall mean of 5.58 ± 0.31. *Riva Light Cure HV* displayed a comparable pattern, with pH values between 5.12 ± 0.04 and 6.03 ± 0.03, averaging 5.68 ± 0.31. *Fuji IX GP Fast* recorded the highest pH values among the materials tested, varying between 5.42 ± 0.07 and 6.23 ± 0.04, with a mean value of 5.93 ± 0.29.

In a neutral environment (pH = 6.8), minimal variations were observed (Figure 1). However, unlike the acidic condition, an initial decrease of approximately one pH unit was detected within the first day, followed by stabilization. For the *Cention Forte Filling Material*, pH values ranged from 5.61 ± 0.08 at 44 °C after 1 day to 6.95 ± 0.08 at 37 °C after 7 days, with an average of 6.33 ± 0.50. A comparable trend was observed for *Riva Light Cure HV*, with pH values spanning from 5.70 ± 0.07 at 44 °C after 1 day to 7.10 ± 0.06 at 37 °C after 7 days, yielding an average of 6.45 ± 0.52. *Fuji IX GP Fast* demonstrated pH levels between 5.90 ± 0.07 at 44 °C after 1 day and 7.25 ± 0.04 at 37 °C after 7 days, with a mean value of 6.68 ± 0.51.

Under basic conditions (pH = 8.8), the trend resembled that observed in the acidic environment, with an initial reduction of approximately two pH units within the first day, followed by a gradual return toward the initial values (Figure 1). The *Cention Forte Filling Material* exhibited pH levels ranging from 6.85 ± 0.04 at 44 °C after 1 day to 8.23 ± 0.04 at 44 °C after 28 days, with an overall mean of 7.70 ± 0.52. Similarly, *Riva Light Cure HV* fluctuated between 6.93 ± 0.08 at 44 °C after 1 day and 8.39 ± 0.07 at 44 °C after 28 days, with an average of 7.82 ± 0.53. *Fuji IX GP Fast* presented pH values from 7.28 ± 0.04 at 44 °C after 1 day to 8.67 ± 0.05 at 44 °C after 28 days, with a mean of 8.08 ± 0.51.

Overall, despite minor differences among the tested materials, the general patterns remained consistent. Under acidic conditions, all materials exhibited an approximate increase of one pH unit, whereas in neutral and basic environments, an initial decrease in pH was observed, followed by stabilization or a gradual return to baseline values.

### 3.2. Fluoride Release

**Figure 2:**
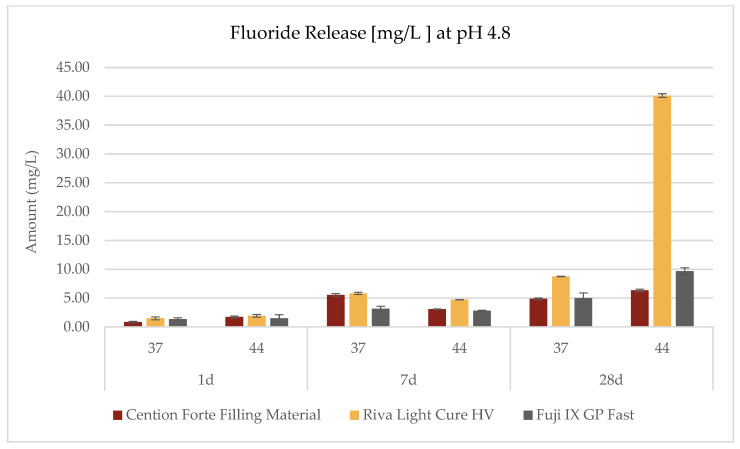
Fluoride release (mg/L) from the *Cention Forte Filling Material*, *Riva Light Cure HV*, and *Fuji IX GP Fast* measured at pH 4.8 after 1, 7, and 28 days and at two temperatures (37 °C and 44 °C).

**Figure 3:**
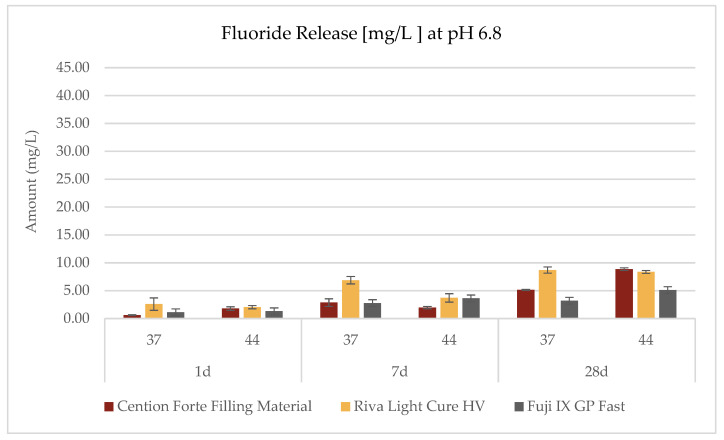
Fluoride release (mg/L) from the *Cention Forte Filling Material*, *Riva Light Cure HV*, and *Fuji IX GP Fast* measured at pH 6.8 after 1, 7, and 28 days and at two temperatures (37 °C and 44 °C).

**Figure 4:**
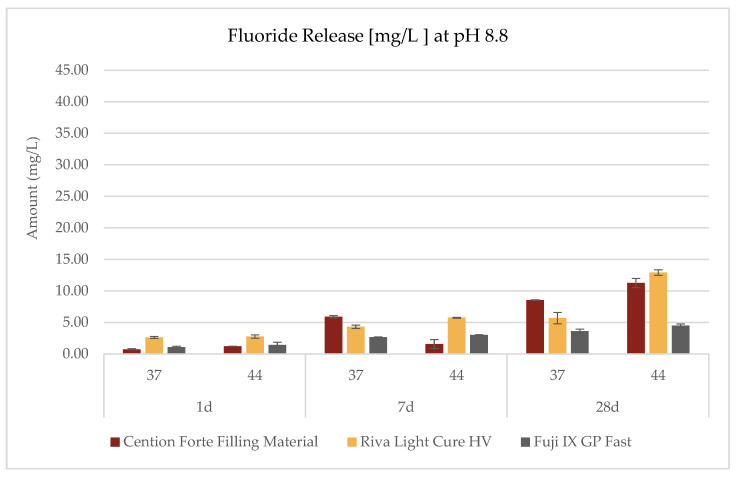
Fluoride release (mg/L) from the *Cention Forte Filling Material*, *Riva Light Cure HV*, and *Fuji IX GP Fast* measured at pH 8.8 after 1, 7, and 28 days and at two temperatures (37 °C and 44 °C).

The investigation into the release profiles of fluoride was conducted on all three materials under three distinct pH conditions, two temperature settings (44 °C and 37 °C), and three observation intervals (1 day, 7 days, and 28 days), as delineated in the accompanying Table 3 and Figure 2, Figure 3 and Figure 4. The data revealed that the fluoride release phenomena are modulated by the interplay of environmental pH, temperature, and exposure duration, with each material exhibiting its own characteristic pattern.

For the *Cention Forte Filling Material*, fluoride release exhibited a progressive increase correlated with prolonged observation periods, elevated temperatures, and higher pH values (Figure 2). Notably, the maximal concentration, 11.25 ± 0.71 mg/L, was attained after 28 days at 44 °C in an alkaline medium (pH = 8.8). Under acidic conditions (pH = 4.8), the observed fluoride concentrations spanned from 0.85 ± 0.13 to 6.35 ± 0.22 mg/L, with an average value of 3.75 ± 2.20 mg/L. In a neutral medium (pH = 6.8), the range extended from 0.61 ± 0.07 to 8.85 ± 0.25 mg/L (mean 3.54 ± 3.01 mg/L), while in an alkaline environment, values ranged from 0.74 ± 0.09 to 11.25 ± 0.71 mg/L (mean 4.87 ± 4.39 mg/L).

In contrast, the *Riva Light Cure HV* material demonstrated an overall superior fluoride release compared to the *Cention Forte Filling Material*. Remarkably, the highest fluoride concentration recorded was 40.14 ± 0.32 mg/L after 28 days under acidic conditions (pH = 4.8) at 44 °C (Figure 3). Specifically, cumulative fluoride release in an acidic medium varied between 1.51 ± 0.25 and 40.14 ± 0.25 mg/L (mean 10.43 ± 14.79 mg/L), whereas at neutral pH the range was 2.04 ± 0.29 to 8.69 ± 0.33 mg/L (mean 5.38 ± 2.96 mg/L), and in an alkaline environment it spanned from 2.63 ± 0.16 to 12.91 ± 0.44 mg/L (mean 5.68 ± 3.79 mg/L).

For the *GC Fuji IX GP Fast* material, the apex of fluoride release was observed under acidic conditions at 44 °C after 28 days, with a peak concentration of 9.69 ± 0.57 mg/L (Figure 4). In this case, the cumulative fluoride release in an acidic environment ranged from 1.35 ± 0.26 to 9.69 ± 0.57 mg/L (mean 3.92 ± 3.12 mg/L), in a neutral environment from 1.13 ± 0.19 to 5.10 ± 0.25 mg/L (mean 2.85 ± 1.49 mg/L), and under alkaline conditions from 1.07 ± 0.14 to 4.48 ± 0.28 mg/L (mean 2.71 ± 1.29 mg/L).

Statistical analyses unequivocally demonstrated that fluoride release is significantly influenced by the acidity of the medium (*p* = 0.009) and exposure duration (*p* < 0.001), with a pronounced tendency for higher fluoride concentrations under conditions of pH-induced stress, whether acidic or alkaline, particularly over extended periods. Multivariate analysis further corroborated the critical influence of both exposure time and pH, revealing statistically significant differences among the materials (*p* < 0.05). Such findings underscore the necessity of accounting for environmental conditions when selecting pediatric restorative materials, given that their ion release behavior—and, by extension, their long-term structural integrity—may be markedly affected by variations in pH and temperature within the oral cavity.

In conclusion, the fluoride release dynamics of the investigated pediatric dental restorative filling materials exhibit distinct, condition-dependent profiles. While the *Cention Forte Filling Material* manifested a consistent, time- and temperature-driven augmentation in fluoride liberation concomitant with increasing pH levels, *Riva Light Cure HV* was characterized by an accentuated release in both acidic and alkaline settings. In fact, the most critical release, indicative of potential material degradation, was observed under acidic conditions (pH 4.8) at high temperature (44 °C), where *Riva Light Cure HV* achieved a maximum release of 40.14 mg/L after 28 days. These results suggest that the performance and durability of restorative materials may be compromised in severe pH environments, thus necessitating careful consideration in their clinical application.

## 4. Discussion

In pediatric dentistry, preserving primary teeth until natural exfoliation is a primary objective, requiring restorative treatment able to resist wear, bacterial activity, and environmental variations such as pH and temperature fluctuations while maintaining aesthetic integrity [33].

Modern restorative approaches aim not only to repair structural damage but also to enhance the resilience of dental tissues against further demineralization [34,35,36,37]. Resin-based dental filling composites have been implicated in facilitating bacterial proliferation at the marginal interfaces of restorations because of debonding and leakage, increasing the risk of secondary caries [38]. This concern has driven the advancement of non-invasive and micro-invasive strategies aimed at enhancing the antimicrobial properties of restorative materials. In this context, bioactive restorative materials have been developed to dynamically release therapeutic ions—fluoride and calcium mainly—with the objective of inhibiting bacterial colonization and biofilm formation on their internal or marginal cavity surfaces, thereby contributing to a more cariostatic and remineralizing environment [22].

This in vitro study examined the kinetics of fluoride release of three restorative materials—*Riva Light Cure HV*, *Fuji IX GP Fast*, and the *Cention Forte Filling Material*—under varying pH and temperature conditions. Given the crucial role of fluoride in the prevention and management of dental caries in pediatric patients, the findings of this study could provide valuable indications, thanks to fluoride release, into the clinical applicability of these materials. Although the experimental design employed in this study follows established protocols for evaluating fluoride release, the research presents a significant contribution by offering a direct and standardized comparative analysis of three widely used pediatric restorative materials: *Riva Light Cure HV*, *Fuji IX GP Fast*, and the *Cention Forte Filling Material*.

Rather than focusing solely on material characterization or ion release in isolation, this study specifically assesses how these materials behave under controlled yet clinically relevant variations in environmental pH and temperature. Such variables mimic conditions commonly encountered in the pediatric oral environment, where dietary habits, oral hygiene, and bacterial metabolism can cause significant fluctuations.

The clinical approach to carious lesions in deciduous dentition presents unique challenges due to the thinner enamel and dentin layers, which render them more susceptible to rapid lesion progression [39]. The application of fluoride-releasing restorative materials in these cases is crucial, as they provide a continuous supply of fluoride, which can facilitate the remineralization of demineralized dentin and enamel [24,40]. Furthermore, given the high prevalence of early childhood caries (ECC), particularly in children under three years of age, the use of fluoride-releasing materials plays a fundamental role in both treatment and preventive strategies. The inclusion of fluoride in restorative materials has been explored for its potential to reduce recurrent caries. Research suggests that fluoride-releasing materials may decrease bacterial adhesion and prevent demineralization at the margins of restorations. Comparative studies indicate that restorations incorporating fluoride exhibit lower rates of secondary caries compared to non-fluoride-releasing alternatives, particularly in patients with elevated caries risk [41,42,43,44].

The findings of this study indicate that pH significantly influenced ion release in all the tested materials, with the highest fluoride release observed under acidic conditions (pH 4.8). This effect, particularly noted in *Riva Light Cure HV*, suggests that lower pH levels facilitate material dissolution, thereby promoting ion release. A substantial fluoride release was observed (40.14 ± 0.32 mg/L) at pH 4.8, after 28 days, highlighting its potential role in supporting enamel remineralization and caries prevention over time, especially in young patients with decayed teeth, where an acidic environment existed because of bacterial metabolism.

Temperature is another critical variable that influences the ionic release of bioactive materials, with studies showing an increase in ion diffusion, including fluoride, at higher temperatures [19,45]. In the oral cavity, especially in children, temperature fluctuations occur with the consumption of hot foods and beverages, influencing material behavior. This study evaluated the effect of a temperature of 44 °C, representing clinically relevant conditions, and found an increase in fluoride release, particularly in acidic conditions (pH 4.8). Notably, *Riva Light Cure HV* exhibited the highest fluoride release under both acidic conditions (pH 4.8) and elevated temperature (44 °C), indicating a possible synergy between these factors in promoting ion release. These findings suggest that temporary exposure to low pH and elevated temperatures may enhance the fluoride release from bioactive materials, thereby potentially increasing their protective effect against demineralization. The positive behavior of this filling dental material, as plotted in Figure 3, could be attributed to its composition. In fact, *Riva Light Cure HV* is described as a light-curing rmGIC, where the polymeric composition is mainly based on 20% HEMA, a well-known hydrophilic resin monomer (Table 2); it strongly differs from the other light-curing alkasite filling dental material, where there are UDMA monomers until 50% in weight. DCP (tricyclodecane dimethanol dimethacrylate) and PEG-400 DMA (polyethylene glycol dimethacrylate), which are interconnected during the light-cure reaction, are the other organic resin components. But UDMA is a linear monomer (Table 2) and a hydrophobic monomer, which probably affects the setting phase and the lower percentage of fluoride release, creating a cross-linked hydrophobic polymer made of monomer chains that is probably able to trap the fluoride diffusion from the Ca-fluorosilicate glass composition of the *Cention Forte Filling Material*. Our findings agree with the results of Ramos et al. (2024) [46]. On the other hand, looking at the Fuji IX fluoride results, something different was scored. As previously suggested by Kelić et al. (2020) [47], this dental filling material is based on a different chemistry. It is, in fact, an acid–base reaction material where resin is missing and setting needs minutes and not seconds; this reaction also occurs in the light-curing dental filling materials compared in this research. In our study, long-term (28 days) fluoride release confirmed high values, but not so high if compared to Kelić et al., who investigated the fluoride release from uncoated samples at 168 days [47].

Furthermore, this study demonstrated that fluoride release increased over time, with the highest cumulative values recorded at 28 days. This prolonged release profile is advantageous in maintaining a sustained anti-cariogenic effect. However, this study also revealed that temperature fluctuations influence fluoride release, with higher temperatures generally leading to increased release. This is a critical consideration, as intraoral temperatures vary depending on dietary intake and physiological factors. So, the null hypothesis of this study was rejected.

One of the major strengths of this study is its controlled in vitro design, which allowed for precise measurement of fluoride release under standardized conditions. The use of advanced analytical techniques, such as ion chromatography, provided accurate quantification, enhancing the reliability of the results. Additionally, the study design accounted for variations in pH and temperature, factors that closely mimic the dynamic oral environment.

In addition, the in vitro nature of this study presents inherent limitations. The absence of salivary flow, enzymatic activity, and complex biofilm interactions may limit the extrapolation of results to in vivo conditions [48]. Therefore, while the findings provide valuable insights, additional in vivo and long-term studies are necessary to fully assess the clinical effectiveness of these restorative materials. Following the initial setting phase, ion release generally declines within 1 day due to polymer network cross-linking, which progressively restricts further ionic diffusion. The findings of this study indicate that bioactive materials exhibited a sustained release of therapeutic fluoride throughout the observation period, highlighting their potential to offer prolonged protection against demineralization while continuously supporting enamel remineralization.

In addition, the findings demonstrate distinctive fluoride release profiles for each material, allowing clinicians to make evidence-based decisions when selecting restorative materials tailored to the individual needs of pediatric patients, particularly those at high risk for recurrent caries. For instance, the notably higher fluoride release exhibited by *Riva Light Cure HV* under acidic conditions suggests a specific clinical advantage in managing children with high caries activity, where a persistently low pH environment is present. Thus, while the methodological novelty is limited, the clinical relevance of the comparative data strengthens this study’s contribution to optimizing restorative strategies in pediatric dentistry.

It should be noted that this study did not assess the solubility of the tested materials. Since solubility can influence both the extent of fluoride release and the long-term structural stability of restorations, future research should incorporate standardized solubility testing to complement ion release data and better predict clinical performance.

Finally, while the basic pH (8.8) used in this study aimed to simulate alkaline oral conditions related to salivary buffering or alkaline oral care products, future investigations using extremely low pH values, such as pH 2.3 in accordance with ISO 22674 [49] could offer further clinically relevant insights into material degradation under severe acidic stress.

Clinically, the results underscore the importance of material selection based on patient-specific conditions. For high-caries-risk pediatric patients, materials with superior fluoride release, such as *Riva Light Cure HV*, may offer enhanced protection against caries progression. Conversely, in cases where mechanical strength is a primary concern, hybrid materials incorporating both fluoride release and improved mechanical resilience should be considered.

## 5. Conclusions

This in vitro study demonstrated that fluoride release from pediatric restorative materials is significantly influenced by environmental pH, temperature, and exposure time. Among the tested materials, *Riva Light Cure HV* exhibited the highest fluoride release, particularly under acidic conditions, suggesting its potential suitability for high-caries-risk pediatric patients. The *Cention Forte Filling Material* and *Fuji IX GP* Fast also showed favorable fluoride release profiles, although to a lesser extent. These results underline the importance of selecting restorative materials based not only on mechanical properties but also on their ability to contribute to caries prevention through sustained fluoride release. Future clinical studies are needed to validate these findings under in vivo conditions.

## Figures and Tables

**Table 1:** Classification and chemical composition of the tested materials based on the manufacturers’ available data (UDMA = urethane dimethacrylate; DCP = Dicyclopropylidene; PEG = polyethylene glycol; DMA = dimethylamine; HEMA = 2-hydroxyethyl methacrylate; GDMA = Glycidyl Methacrylate; DMAEMA = N, N-Dimethylaminoethyl Methacrylate; and EDMAB = Ethyl Dimethyl Amino Benzoate).

Material	Manufacturer	Type	Curing Mechanism	Composition
**Cention Forte Filling** **Material** (Lot n. ZL08SR)	Ivoclar (Schaan, Liechtenstein)	Alkasite	Self-curing with a light-curing option	Ca-fluorosilicate glass, Ba-Al silicate glass, copolymer, Ca-Ba-Al-fluorosilicate glass, 25–50% UDMA, 2.5–10% ytterbium trifluoride, 10–25% aromatic aliphatic UDMA, DCP, and PEG-400-DMA
**Fuji IX GP Fast** (Lot n. 2311271)	GC Corp (Tokyo, Japan)	Glass ionomer	Self-setting	Alumino-silicate glass, 25 ≤ 50% Polyacrylic acid, water, and 5 ≤ 10% tartaric acid
**Riva Light Cure HV** (Lot n. 1237227)	SDI (Victoria, Australia)	High-viscosity glass ionomer	Light-curing	10–20% HEMA, 10–20% HEMA-phosphate derivative, 1–10% GDMA, 1–7% DMAEMA, 1–5% tartaric acid, 0–1% EDMAB, 0–1% camphorquinone, 0–1% BHT, >90% glass, oxide, and 1–10% silica amorphous

**Table 2:** Mean pH values and corresponding standard deviations (SDs) of the soaking solutions following the immersion of each tested material in acidic, neutral, and basic environments, measured at two temperatures (37 °C and 44 °C) over three observation periods (1 day, 7 days, and 28 days).

Buffer Solution pH	Time	T (°C)	Cention Forte Filling	Riva Light Cure	Gc Fuji IX GP Fast
4.8	1 day	44	5.70 ± 0.04	5.80 ± 0.08	6.15 ± 0.03
		37	5.02 ± 0.06	5.12 ± 0.04	5.42 ± 0.07
	7 days	44	5.9 1± 0.07	6.03 ± 0.03	6.23 ± 0.04
		37	5.75 ± 0.03	5.85 ± 0.06	6.07 ± 0.06
	28 days	44	5.59 ± 0.04	5.74 ± 0.07	5.85 ± 0.05
		37	5.53 ± 0.04	5.64 ± 0.05	5.86 ± 0.03
6.8	1 day	44	5.61 ± 0.08	5.70 ± 0.07	5.90 ± 0.07
		37	5.90 ± 0.07	6.02 ± 0.08	6.31 ± 0.03
	7 days	44	6.31 ± 0.07	6.41 ± 0.05	6.64 ± 0.06
		37	6.95 ± 0.08	7.10 ± 0.06	7.25 ± 0.04
	28 days	44	6.53 ± 0.03	6.66 ± 0.07	6.97 ± 0.03
		37	6.67 ± 0.05	6.80 ± 0.06	7.03 ± 0.05
8.8	1 day	44	6.85 ± 0.04	6.93 ± 0.08	7.28 ± 0.04
		37	7.36 ± 0.04	7.5 ± 0.03	7.73 ± 0.04
	7 days	44	8.06 ± 0.07	8.15 ± 0.05	8.44 ± 0.06
		37	8.02 ± 0.05	8.11 ± 0.04	8.30 ± 0.07
	28 days	44	8.23 ± 0.04	8.39 ± 0.07	8.67 ± 0.05
		37	7.68 ± 0.06	7.86 ± 0.03	8.09 ± 0.03

**Table 3:** Mean fluoride concentrations along with standard deviations (SDs) recorded for each tested material under three distinct pH conditions (4.8, 6.8, and 8.8), measured at two temperatures (37 °C and 44 °C) and across three observation periods (1 day, 7 days, and 28 days).

Buffer Solution pH	Time	T (°C)	Fluoride Release [mg/L]		
			Cention Forte Filling Material	Riva Light Cure HV	Fuji IX GP Fast
4.8	1 day	37	0.85 ± 0.13	1.51 ± 0.25	1.35 ± 0.26
		44	1.73 ± 0.17	1.95 ± 0.23	1.5 ± 0.63
	7 days	37	5.56 ± 0.24	5.84 ± 0.19	3.17 ± 0.44
		44	3.09 ± 0.05	4.73 ± 0.02	2.81 ± 0.08
	28 days	37	4.89 ± 0.14	8.76 ± 0.03	5.00 ± 0.9
		44	6.35 ± 0.22	40.14 ± 0.32	9.69 ± 0.57
6.8	1 days	37	0.61 ± 0.07	2.59 ± 1.11	1.13 ± 0.19
		44	1.79 ± 0.32	2.04 ± 0.29	1.30 ± 0.42
	7 days	37	2.86 ± 0.69	6.87 ± 0.66	2.77 ± 0.71
		44	1.97 ± 0.21	3.71 ± 0.76	3.61 ± 0.25
	28 days	37	5.14 ± 0.08	8.69 ± 0.33	3.20 ± 0.33
		44	8.85 ± 0.25	8.36 ± 0.26	5.10 ± 0.25
8.8	1 day	37	0.74 ± 0.09	2.63 ± 0.16	1.07 ±0.14
		44	1.23 ± 0.01	2.76 ± 0.26	1.44 ± 0.41
	7 days	37	5.89 ± 0.19	4.31 ± 0.27	2.65 ± 0.05
		44	1.58 ± 0.7	5.75 ± 0.07	3.00 ± 0.07
	28 days	37	8.55 ± 0.04	5.69 ± 0.91	3.63 ± 0.3
		44	11.25 ± 0.71	12.91 ± 0.44	4.48 ± 0.28

## Data Availability

The original contributions presented in this study are included in the article; further inquiries can be directed to the corresponding author.
